# A Symmetric ICA-Based CDMA Receiver for Dense IoT-Enabled Healthcare Monitoring Systems

**DOI:** 10.3390/s26144501

**Published:** 2026-07-15

**Authors:** Muhammad Irfan Anjum, Abdullah Waqas, Asad Saleem

**Affiliations:** 1 School of Electronic Engineering and Intelligent Manufacturing, Anhui Xinhua University, Hefei 230088, China; 2 Faculty of IT, University of Central Punjab, Lahore 54000, Pakistan; irfan.anjum@ucp.edu.pk; 3 Department of Electrical Engineering, National University of Technology, Islamabad 44000, Pakistan; abdullah@nutech.edu.pk

**Keywords:** IoT-enabled healthcare, wearable medical sensors, multiuser interference mitigation, Independent Component Analysis, real-time multiuser detection, remote patient monitoring, Higher-order Statistics

## Abstract

The rapid development of Internet of Things (IoT)-enabled healthcare systems, including wearable medical sensors and remote patient monitoring devices, has led to dense multiuser communication scenarios in which numerous low-power devices simultaneously transmit physiological data. In such environments, multiuser interference significantly degrades reliability and increases error rates, which may compromise clinical decision-making. This paper proposes a real-time symmetric Independent Component Analysis (ICA)-based Code Division Multiple Access (CDMA) receiver, termed symmetric independent CDMA (i-CDMA) receiver, to enhance multiuser detection in dense healthcare IoT networks. Unlike conventional ICA-CDMA receivers that utilize the orthogonality of users’ spreading codes and higher-order statistics (HoS) of users’ message symbols in signal separation, the proposed approach utilizes a symmetric independence process that jointly utilizes the independence of separating codes and the statistical independence of users’ message symbols. The proposed receiver avoids the runtime optimization of scaling parameter for different user densities and SNR conditions. Furthermore, a modified whitening transform is introduced to exploit the complete signal-plus-noise subspace while avoiding overlearning. Simulation results demonstrate significant performance improvement over symmetric Gram–Schmidt orthogonalization-based standard ICA-CDMA receiver in terms of bit error rate (BER) under varying SNR and user-density scenarios for both uplink and downlink systems. The proposed receiver provides a scalable and interference-resilient communication framework suitable for real-time IoT-enabled healthcare monitoring applications.

## 1. Introduction

The rapid growth of Internet of Medical Things (IoMT)-enabled healthcare systems has transformed modern patient monitoring and clinical data acquisition [1,2,3]. Wearable medical sensors, implantable devices, and remote healthcare monitoring platforms continuously transmit physiological information such as electrocardiogram (ECG), oxygen saturation, blood pressure, and glucose levels to centralized monitoring systems. In dense healthcare environments including smart hospitals and continuous patient observation systems, a large number of low-power medical devices may operate simultaneously within limited spectral resources, resulting in significant multiuser interference (MAI), transmission unreliability, and increased error rates [4].

Reliable and interference-resilient communication is therefore essential for accurate clinical decision-making in IoMT systems [5,6]. Recent studies have highlighted the increasing communication challenges associated with scalable healthcare infrastructures. Jin et al. [7] emphasized lightweight cryptographic protection mechanisms for medical IoT systems, while Zhao et al. [8] demonstrated the importance of dependable communication infrastructure in blockchain-enabled healthcare architectures. Similarly, Hu et al. [9] showed that wearable smart-textile sensors operate under strict power and computational constraints, requiring energy-efficient and interference-resilient communication techniques. More recently, Xiao et al. [10] and Xu et al. [11] investigated learning-based interference mitigation and federated healthcare communication frameworks for robust distributed healthcare systems.

To achieve this requisite communication efficiency in modern deployments, contemporary medical IoT infrastructures have increasingly looked toward advanced index-modulation and chaotic-transmission paradigms to protect telemetry under tight energy budgets rather than relying solely on legacy code-division multiple access (CDMA). Recent architectures have successfully implemented joint index and spread-spectrum approaches, specifically leveraging energy-efficient continuous phase modulation (CPM) tailored for low-power IoT networks [12], alongside advanced multi-carrier initial-condition-index-aided differential chaos shift keying (DCSK) frameworks optimized for high-efficiency, secure wireless links [13]. While these emerging low-power modulation and index-based multiple-access paradigms provide excellent energy scalability for wide-area networks, the proposed symmetric i-CDMA framework functions as a highly complementary, high-throughput solution. It is specifically optimized for dense, localized, and continuous physiological monitoring environments (e.g., intensive care units) where massive code reuse, ultra-low latency, and blind multiuser separation are strictly required at the physical layer.

Traditional distributed architectures within these localized, high-density environments continue to find strong utility in spread-spectrum paradigms. Specifically, Direct Sequence Code Division Multiple Access (DS-CDMA) serves as a flexible multiuser communication technique that enables multiple low-power devices to simultaneously transmit over a shared wireless channel using distinct spreading codes. Due to its architectural scalability and inherent interference resilience, DS-CDMA remains a cornerstone candidate for dense wireless sensor and IoMT healthcare deployments. However, the simultaneous transmission of multiple users over identical spectral resources inevitably introduces multiuser interference (MUI) or multiple access interference (MAI), which severely degrades system performance under dense user conditions [14]. Conventional CDMA receivers, such as the matched filter and minimum mean square error (MMSE) detectors, primarily rely on second-order statistics (SoS) and the strict orthogonality of spreading codes for signal separation—limiting their effectiveness in highly interfered or fading-heavy environments.

To overcome these structural limitations and improve multiuser detection performance, Independent Component Analysis (ICA)-based blind CDMA receivers have been extensively investigated. Early studies by Cristescu et al., Ekici et al., El-Khamy et al., and Ristaniemi et al. [15,16,17,18,19] demonstrated that higher-order statistics can effectively enhance blind multiuser detection without requiring explicit channel estimation.

Subsequent works further extended ICA-CDMA frameworks using FastICA [17], adaptive ICA, JADE [20], RADICAL ICA [21], and hybrid blind detection techniques. Compared with conventional SoS-based receivers, ICA-CDMA approaches provide improved interference suppression by exploiting statistical independence among user signals [22].

Despite these improvements, existing ICA-CDMA receivers suffer from several important limitations. Many ICA-based approaches rely on parameterized nonlinear contrast functions whose performance is sensitive to initialization, learning rate selection, and nonlinearity control parameters [23]. In practical IoMT healthcare environments, where user density and communication conditions may vary dynamically, such parameter sensitivity may lead to unstable convergence and degraded detection performance. Furthermore, several ICA-CDMA receivers employ deflationary estimation, where separating vectors are updated sequentially [24]. In such approaches, estimation errors introduced in early stages may propagate to subsequent separating vectors, reducing robustness in dense multiuser scenarios. Existing methods may also require auxiliary information, including channel estimation, multipath delay estimation, and denoising preprocessing. These additional requirements increase computational complexity and implementation overhead.

Recent communication paradigms including non-orthogonal multiple access (NOMA), MIMO-based detection, and learning-based receivers have introduced alternative approaches for interference mitigation in dense wireless systems [25]. NOMA improves spectral efficiency through power-domain multiplexing [26], while MIMO-based techniques exploit spatial diversity to improve separation performance [27]. Similarly, learning-based receivers utilize data-driven optimization for adaptive interference suppression [28]. Although these methods provide promising performance gains, they generally require accurate channel state information, extensive training data, or high computational resources, which may limit their applicability in resource-constrained IoMT healthcare devices.

Beyond advances in communication technologies, recent studies have highlighted the importance of comprehensive IoMT architectures that integrate wearable medical devices, wireless body area networks (WBANs), edge computing, cloud services, and intelligent healthcare applications to support continuous patient monitoring and timely clinical intervention. Pazienza et al. [29] proposed an adaptive critical care intervention framework that combines wearable sensing devices, edge intelligence, continuous monitoring, and clinical risk assessment to enable early warning and personalized healthcare services. Such IoMT systems impose stringent communication requirements, including high reliability, low latency, scalability, and robust interference mitigation, since physiological signals such as ECG, SpO_2_, blood pressure, and body temperature must be transmitted accurately and continuously from multiple devices. Although the present work focuses on physical-layer multiuser detection rather than healthcare analytics, the proposed symmetric ICA-based CDMA receiver contributes to these IoMT architectures by providing reliable interference suppression and improved detection performance in dense healthcare communication environments, thereby supporting dependable transmission of critical medical information.

Furthermore, contemporary medical IoT deployments have increasingly looked toward alternative low-power wide-area network (LPWAN) protocols and chaotic modulations to protect data transmission under tight energy budgets. Recent architectures have successfully implemented secure and lightweight tracking protocols tailored for LoRa-based low-power sensor networks, alongside advanced multi-input multi-output differential chaos shift keying (MIM-DCSK) modulation frameworks optimized for high-reliability green IoMT applications. While these emerging multiple-access and modulation paradigms provide excellent wide-area scalability, the proposed symmetric i-CDMA framework functions as a highly complementary, high-throughput solution specifically optimized for dense, localized, and continuous physiological monitoring environments (e.g., intensive care units) where dense code reuse and blind multiuser separation are heavily required.

Table 1 highlights the principal differences between the proposed symmetric i-CDMA receiver and previously reported ICA-CDMA variants. Unlike existing approaches, the proposed framework simultaneously updates all separating vectors, eliminates error propagation, avoids runtime parameter tuning, and exploits the complete signal-plus-noise subspace [30].

Therefore, there remains a need for a lightweight and scalable blind multiuser detection framework that reduces parameter sensitivity, avoids error propagation, and maintains stable convergence without requiring extensive training or adaptive parameter tuning.

Motivated by these challenges, this paper proposes a symmetric independence-based ICA-CDMA receiver, termed symmetric i-CDMA receiver, for dense IoMT-enabled healthcare communication environments. Unlike conventional deflationary ICA-CDMA receivers, the proposed framework employs a symmetric update mechanism that jointly refines all separating vectors, thereby reducing error propagation and improving convergence stability. In addition, a modified whitening transformation is introduced to preserve the complete signal-plus-noise subspace, enabling improved extraction of statistically independent separating vectors while avoiding overlearning effects associated with conventional approaches.

The main contributions of this work are summarized as follows:

- A symmetric independence-based ICA-CDMA receiver that eliminates sequential error propagation associated with deflationary ICA methods;
- A modified whitening transformation that preserves the complete signal-plus-noise subspace for enhanced separation performance;
- A coupled optimization framework utilizing matrices W and L for stable estimation of statistically independent separating vectors;
- Comprehensive performance evaluation under varying SNR and user-density conditions for both CDMA uplink and downlink systems.

The remainder of this paper is organized as follows. Section 2 presents the system model. Section 3 reviews the standard ICA-CDMA receiver. Section 4 introduces the proposed symmetric i-CDMA receiver and complexity analysis. Section 5 presents the simulation results and discussion, while Section 6 concludes the paper.

## 2. System Model

We consider a dense IoT healthcare monitoring environment where *K* wearable medical sensors simultaneously transmit physiological data (e.g., ECG, SpO_2_, and blood pressure) to a central monitoring unit. If users are assumed to be completely synchronous, the composite transmitted base-band signal $D\left(t\right)$ for *K* users can be written as [33]

(1) $$D\left(t\right)=\sum_{k=1}^{K}\sum_{m=1}^{M}b_{k}\left(m\right)s_{k}\left(t-mT_{b}\right),$$

where $b_{k}\left(m\right)$ denotes the *m*-th symbol of the *k*-th user, $s_{k}\left(.\right)$ represents spreading code of the *k*-th user, and *M* is the number of binary message symbols. Each code has a chip duration $T_{c}$, while the bit duration of each message symbol is $T_{b}$, where $T_{b}\gg T_{c}$. For the AWGN channel, the received base-band signal can be written as

(2) $$x\left(t\right)=\sum_{k=1}^{K}\sum_{m=1}^{M}b_{k}\left(m\right)s_{k}\left(t-mT_{b}\right)+n\left(t\right),$$

where $n\left(t\right)$ represents additive white Gaussian noise (AWGN) noise. The received signal $x\left(t\right)$ is a mixture of *K* user signals corrupted by additive noise. The objective at the receiver is to separate the message signal of an intended user from this corrupted mixture using the spreading code for that particular user. Continuous-time received signal (Equation (2)) is integrated over one chip duration using normalized chip gain matched filter. The result of this integrator is then sampled at the chip rate to yield the following discrete-time signal [34]:

(3) $$x\left[mT_{c}\right]=\frac{1}{T_{c}}\int_{mT_{c}}^{\left(m+1\right)T_{c}}x\left(t\right)dt.$$

The chip-matched filter sampled output (Equation (3)) is collected into a [C×1] dimension vector $x\left(m\right)$,

(4) $$x\left(m\right)={\left[x\left(mC\right)T_{c},\;x\left(mC+1\right)T_{c}\;\cdots ,\;x\left(mC+C-1\right)T_{c}\right]}^{T}.$$

The received vector $x\left(m\right)$ contains the information of *C* received chips corresponding to *m*-th symbol of all users and has the form [34]

(5) $$x\left(m\right)=\sum_{k=1}^{K}\left[b_{k}\left(m\right)s_{k}\right]+n\left(m\right),$$

where $s_{k}={\left[s_{k}\left(0\right)\;s_{k}\left(1\right)\;\cdots ,\;s_{k}\left(C-1\right)\right]}^{T}$ is the *k*-th user’s spreading code vector and $n\left(m\right)={\left[n\left(0\right)\;n\left(1\right)\;\cdots ,\;n\left(C-1\right)\right]}^{T}$ denotes the noise vector in the *m*-th received signal sample. More compactly, Equation (5) can be represented in matrix-vector notation as

(6) $$x\left(m\right)=\mathrm{Gb}\left(m\right)+n\left(m\right),$$

where $\left[C\times K\right]$ dimensional matrix $G=\left[s_{1},\;s_{2}\;\ldots ,\;s_{K}\right]$, consisting of all the codes, is assumed to be full-rank, and the *K* dimensional vector $b\left(m\right)={\left[b_{1}\left(m\right),b_{2}\left(m\right),\ldots ,b_{K}\left(m\right)\right]}^{T}$ contains the *m*-th received symbol of *K* users. The matrix form representation of the received signal for *M* message symbols for all users can be written as [35]

(7) X=GB+N,

where

$$X=\left[x\left(1\right)\;x\left(2\right)\;\ldots ,\;x\left(M\right)\right],$$

$$B=\left[b\left(1\right)\;b\left(2\right)\;\ldots ,\;b\left(M\right)\right],$$

and

$$N=\left[n\left(1\right)\;n\left(2\right)\;\ldots ,\;n\left(M\right)\right].$$

Here, the received signal matrix X, having $\left[C\times K\right]$ dimensions, contains the mixture of chips from *K* users for *M* symbols. The receiver’s objective is to separate an individual’s received signal at chip rate and extract source symbols by minimizing the mean squared error. Whitening transform on the received signal matrix is performed to bring it one step closer to estimating the source signal. Usually, whitening on the received signal is performed by the eigenvalue decomposition of X

(8) $$QVQ^{T}=E\left[XX^{T}\right],$$

where the $\left[C\times C\right]$ dimension matrices V and Q denote, respectively, the eigenvalue and eigenvector matrices corresponding to the signal-plus-noise subspace. The matrix X denotes the received signal observation matrix composed of the mixtures of transmitted user symbols collected over multiple received samples. Each column of X corresponds to a received observation vector at a particular sampling instant. The expectation operator E{·} represents the statistical expectation computed over the received signal samples and is used to estimate the covariance structure of the observed multiuser signal mixture. In order to filter out excess noise, dimensionality reduction is performed by selecting *K* principal eigenvalues and eigenvectors corresponding to *K* users. The whitening transform can be written as

(9) $$Z=V^{-1/2}Q^{T}X.$$

The matrix Q represents the orthogonal eigenvector matrix obtained from the eigenvalue decomposition of the covariance matrix of the received signal. The columns of Q define the principal orthogonal directions of the received signal space and are used to decorrelate the observed user mixtures during the whitening transformation process. The whitening transform removes the second-order dependence among mixed signal, and the whitened matrix Z is considered as the matrix comprising of decorrelated components having unit variance. The selection of only *K* principal components out of *C* reduces the computation required. During the whitening transform, the primary objective is to achieve decorrelation of the mixed components. Additionally, noise reduction is also achieved by filtering out the noise through the selection of principal components only.

## 3. Standard ICA-Based CDMA Receiver

In dense healthcare IoT environments, conventional ICA-CDMA receivers may experience performance degradation due to parameter sensitivity and error propagation in deflationary orthogonalization method, which can reduce reliability under high user density. In CDMA, the problem of extraction of users’ symbols from the received mixture (Equation (6)) resembles blind source separation. It can be solved using the noisy ICA model given in [36] with the assumption that the mixing matrix G is full rank and non-Gaussian source vector $b\left(m\right)$ is to be determined. To find the independent source vector $b\left(m\right)$, whitening is first performed on the received mixed signal (Equation (9)). Whitening transform decorrelates the mixed components in the received signal and normalizes their variances. Coincidentally, it also reduces the dimension of mixed signal and suppresses additive noise. Once the whitening has been performed, another transformation P is to be determined, which gives estimates of independent source signals, i.e.,

(10) $$\hat{B}=sgn\left[P^{T}Z\right],$$

where P is the separating matrix obtained through the ICA algorithm and $\hat{B}$ denotes the estimated *M* symbols of *K* users. ICA finds this transformation P based on an optimization criterion for the independence of the signals, e.g., maximization of the non-Gaussianity of the separated signals. It can be argued that the distribution of a random variable would be less Gaussian than any (weighted) sum of the independent random variables. Based on the observation, the separation matrix P in Equation (10) can be determined by maximizing the non-Gaussianity of component sources in the whitened mixture Z. Kurtosis, being the fourth-order cumulant of a random variable, can be used as one of the quantitative measures of non-Gaussianity [37]. The kurtosis of a random variable *J* can be defined as

(11) $$kurt\left(J\right)=E\left\{J^{4}\right\}-3{\left(E\left\{J^{2}\right\}\right)}^{2}.$$

For zero mean whitened and normalized random variable, Equation (11) simplifies to $E\left\{J^{4}\right\}-3$, implying that the kurtosis is just a normalized version of the fourth moment $E\left\{J^{4}\right\}$. The optimization of the separating matrix P is usually performed with any of the available stochastic gradient algorithms. The fast fixed-point algorithm, considering the kurtosis as a measure of the non-Gaussianity, can be stated as [38]

(12) $$p_{k}\leftarrow \frac{1}{M}E\left\{Z{\left(Z^{T}p_{k}\right)}^{3}\right\}-3{∥p_{k}∥}^{2}p_{k},$$

where $p_{k}$ is the *k*-th column of separating matrix P and *K* such entries constitute a separating matrix. The cube in the product ${\left(Z^{T}p_{k}\right)}^{3}$ is obtained by raising each element of the resultant vector to the power 3. FastICA is one of the most efficient and effective ICA algorithms because of its fast convergence and computational simplicity. Its modification is presented in [39] for noisy channels. Noisy FastICA has been applied to CDMA communication in [16,18,19,40,41]. ICA algorithms determine each vector of the separating matrix iteratively. After each iteration, the separating matrix vectors $p_{1},p_{2},\ldots p_{K}$, are mutually orthogonalized by the Gram–Schmidt orthogonalization. The mutual orthogonalization between the components of the separating matrix P can be achieved in one of two possible ways. They can either be orthogonalized all at once, called symmetric orthogonalization, or by orthogonalizing one component at a time, known as deflationary orthogonalization. To find the separating matrix P, the FastICA algorithm can be applied in conjunction with any one of these two orthogonalization schemes. In deflationary-based orthogonalization, the vectors $p_{1},p_{2},\ldots p_{K}$, are orthogonalized by Gram–Schmidt orthogonalization [38]

(13) $$p_{k}\leftarrow p_{k}-\sum_{j=1}^{k-1}\langle p_{k},p_{j}\rangle p_{j},$$

where $\langle .,.\rangle$ denotes the inner product in Euclidean space. Generally, the *k*-th separating vector, $p_{k}$, is found by orthogonalizing it with all previously obtained separating vectors. Mutual orthogonalization between these separating vectors is achieved by taking the sum of its inner products with previously calculated separating vectors and subtracting it from the current vector. One problem associated with this type of orthogonalization is that, during the estimation of separating vectors, if an error is induced in one of the vectors, it will propagate in all the remaining vectors. In symmetric orthogonalization-based standard ICA-CDMA receiver, prior to the orthogonalization of separating matrix components, the fixed point FastICA algorithm (Equation (12)) is executed for all users. For *K* users, the update equation (Equation (12)) can be written as

(14) $$P\leftarrow \frac{1}{M}E\left\{Z{\left(Z^{T}P\right)}^{3}\right\}-3{∥P∥}^{2}P.$$

Matrix P corresponds to the *K* users’ separating vectors $p_{1},p_{2},\ldots p_{K}$. These separating vectors can be orthogonalized all at once via symmetric Gram–Schmidt orthogonalization in the following fashion:

(15) $$P\leftarrow \frac{3}{2}P-\frac{1}{2}\left(PP^{T}\right)P.$$

The FastICA algorithm and the orthogonalization on the separating matrix P is performed until the convergence of individual vectors. During the symmetric orthogonalization step, all the separating vectors are calculated simultaneously, reducing the chances of error propagation. In both orthogonalization schemes, after finding the *k*-th user’s separating vector $p_{k}$, the estimate of *k*-th user’s symbols is determined using

(16) $${\hat{b}}_{k}=sgn\left[p_{k}^{T}Z\right].$$

Although mutually orthogonal separating vectors are conventionally guaranteed via Gram–Schmidt orthogonalization in Euclidean space, it is argued that establishing mutual statistical independence among these vectors provides a vital performance margin.

## 4. Independent ICA-Based CDMA Receiver

Symmetric i-CDMA receiver utilizes the independence of codes in addition to the independence of sources by employing the symmetric independence process. In this process, signal-plus-noise subspace is utilized in the extraction of independent separating vectors. Complete subspace (signal-plus-noise) is utilized by modifying the standard whitening transform. To understand the differences in modified whitening transform, recall that the standard whitening transform is performed through the eigenvalue decomposition of the received signal (Equation (8)). Dimensionality reduction is performed by selecting only *K* principal eigenvalues and eigenvectors. It filters out the noise and reduces the required computations. However, due to the dimension reduction, some useful information is discarded. It is argued that the performance can be improved by utilizing the complete subspace (signal-plus-noise) and performing the noise reduction at the same time. This is achieved by utilizing the *K* principal eigenvector matrix Q and eigenvalue matrix V in the modified whitening transform such as

(17) $$Z^{\prime }=QV^{-1/2}Q^{T}X,$$

where the superscript ^′^ on $Z^{\prime }$ is used to distinguish this whitening transform from the standard version (Equation (9)). This $\left[C\times M\right]$ dimensional matrix $Z^{\prime }$ is assumed to be white, having unit variance and containing information corresponding to signal-plus-noise subspace. Although the noise reduction is performed by selecting only the *K* principal components of Q and V, the original subspace (signal-plus-noise) is restored by multiplying the standard whitening product (Equation (9)), with the matrix Q from the left side. This restores the dimensions of the whitened matrix Z from $\left[K\times M\right]$ to $\left[C\times M\right]$ resulting in $Z^{\prime }$.

Unlike conventional whitening approaches that retain only the principal signal subspace, the proposed whitening transformation preserves the complete signal-plus-noise subspace during preprocessing. This modification is motivated by the fact that ICA-based separation relies on higher-order statistical information that may be partially lost during dimensionality reduction. By preserving the complete received signal representation, the proposed framework maintains additional statistical information useful for independent component estimation while still achieving decorrelation and variance normalization of the observed mixtures. This contributes to improved separation stability and interference suppression performance under dense multiuser conditions. Therefore, the proposed whitening formulation avoids excessive dimensionality reduction while preserving the statistical diversity required for stable ICA-based multiuser separation. Compared with the standard whitening transform, the proposed formulation preserves additional dimensions of the received signal space and therefore provides a richer representation for estimating statistically independent separating vectors. To utilize the HoS of sources in user signal separation, the separating matrix W is initialized using the modified whitening transform

(18) $$W=QV^{-1/2}Q^{T}\hat{G},$$

where matrix $\hat{G}$ corresponds to *K* users spreading code matrix, usually defined randomly in CDMA downlink and completely known in the CDMA uplink. The separating matrix W is updated as follows:

(19) $$W\leftarrow \frac{1}{M}E\left\{Z^{\prime }{\left({Z^{\prime }}^{T}W\right)}^{3}\right\}-3{∥W∥}^{2}W.$$

This update equation is similar to the standard symmetric ICA-CDMA receiver (Equation (14)), with the exception that the dimensions of the whitened matrix $Z^{\prime }$ and separating matrix W are enhanced. Column vectors of this matrix correspond to *K* different users and to be able to separate users’ message symbols from the mixture, they need to be transformed so that they become mutually statistically independent. The update operation (Equation (19)) incorporates HoS of the received signal $Z^{\prime }$. To exploit the HoS of separating vectors, it is proposed to apply the standard ICA algorithm on columns of the separating matrix W by perceiving them as received signals. To obtain statistically independent column vectors of W, separating matrix L is introduced. This matrix is updated through the FastICA algorithm till convergence. Once converged, it is used to extract independent columns of W. Matrix W is then utilized to extract independent sources. The estimate of message symbols for *K* users can be found using

(20) $$\hat{B}=sgn\left[W^{T}Z^{\prime }\right].$$

It is worth mentioning here that when the whitening transform (Equation (17)) and initialization (Equation (18)) are utilized directly within a standard, unconstrained ICA-CDMA receiver, the algorithm inherently starts to overlearn and completely fails to converge. This catastrophic divergence occurs because the reintroduction of the complete noise subspace forces the FastICA algorithm to track random noise power spectral spikes, interpreting them as non-Gaussian independent components. The proposed symmetric i-CDMA receiver uniquely overcomes this overlearning limitation through its coupled multi-matrix optimization loop defined by the relation $W={\left[L^{T}Y\right]}^{T}$. Because the auxiliary separating matrix L is strictly initialized using code-space constraints and iteratively refined via symmetric statistical independence bounds, it effectively serves as an un-parameterized, data-driven subspace filter. During each iterative coupled update, multiplying the raw whitened component matrix Y by the mathematically clean matrix L projects the separation vectors cleanly back onto the true signal manifold. This structural projection dynamically isolates and suppresses noise subspace tracking, successfully preventing overlearning while fully exploiting the extended sample ensemble diversity. The detailed description is given subsequent Section 4.1 and Section 4.2. The improvements highlighted are particularly beneficial for IoT-enabled healthcare deployments where stable multiuser detection is required to ensure continuous and reliable monitoring of patient data from wearable medical devices.

### 4.1. Symmetric i-CDMA Uplink System

The proposed symmetric i-CDMA uplink receiver performs multiuser detection by jointly estimating statistically independent components from the received signal. The overall processing consists of three main stages: whitening, symmetric ICA-based separation, and symbol estimation.

Initially, the received signal matrix X is transformed using a modified whitening operation to obtain $Z^{\prime }$. Unlike conventional whitening, which reduces dimensionality by selecting only principal components, the modified whitening transformation preserves the complete signal-plus-noise subspace and performs necessary whitening. This enables the algorithm to exploit additional structural information present in the received mixture while still ensuring decorrelation and unit variance of the transformed signals.

The separation process is performed through two coupled matrices, W and L. The matrix W acts as the primary separating matrix that extracts user signals from the whitened mixture $Z^{\prime }$. Its update is based on a fixed-point formulation that maximizes statistical independence by exploiting higher-order statistics of users’ symbols. The cubic operation appearing in the update equation corresponds to a kurtosis-based contrast function. To further enhance independence among separating vectors, an auxiliary matrix L is introduced. Matrix L acts as a secondary separation stage that enforces statistical independence among the columns of W. The columns of W are interpreted as input signals, and a second ICA operation is applied and matrix L is updated. After each update, L is orthogonalized using a symmetric orthogonalization scheme which prevents mixtures from converging to the same maxima. The updated matrix L is then used to refine W, creating an iterative loop between the two matrices. The iterative updates of W and L are continued until convergence is achieved. Convergence is defined using the relative Frobenius norm as

(21) $$\frac{∥L^{\left(t+1\right)}-L^{\left(t\right)}{∥}_{F}}{∥L^{\left(t\right)}{∥}_{F}}<\epsilon$$

(22) $$\frac{∥W^{\left(t+1\right)}-W^{\left(t\right)}{∥}_{F}}{∥W^{\left(t\right)}{∥}_{F}}<\epsilon$$

In this work, ϵ is set to $10^{-6}$, which provides an optimal balance between symbol estimation accuracy and real-time computational efficiency. Empirically, the convergence trajectories of the coupled Frobenius norms (Equations (21) and (22)) exhibit consistent monotonic decay characteristics across varying operational scenarios. Under low user-density environments (K=7) operating at high-SNR regimes (e.g., 8dB), the matrix trajectories rapidly stabilize and meet the termination threshold within approximately 18 to 25 iterations. Conversely, under worst-case dense multiuser scenarios (K=13) operating at deeply negative SNR thresholds (e.g., −4dB), the inclusion of the noise subspace introduces minor structural fluctuations during the initial 10 iterations; however, the coupled projections successfully steer the trajectories to cross the $\epsilon =10^{-6}$ boundary within 34 to 38 iterations. Thus, setting a hard cap of 40 iterations provides a mathematically reliable upper convergence bound for highly dynamic operating states.

This iterative update of matrices L, and W enforces mutual statistical independence among separating vectors, which in turn improves user signal separation accuracy and stability. The use of a symmetric update structure ensures that all separating vectors are refined simultaneously, thereby eliminating the sequential dependency and error propagation associated with deflationary ICA approaches. Moreover, in this algorithm nonlinearity is fixed. It does not require runtime optimization of nonlinear control parameters unlike the Gram–Schmidt base deflationary i-CDMA receiver [30]. The implementation steps are listed in Algorithm  1.

### 4.2. Symmetric i-CDMA Downlink System Model

In the CDMA downlink, the intended user has the information of its intended code only. Therefore, in standard ICA-CDMA downlink receivers, the separating matrix P is initialized randomly and is updated through the FastICA algorithm (Equation (14)). The *k*-th intended user utilizes its assigned spreading code $s_{k}$ to extract its message symbols from the mixture. Spreading code $s_{k}$ is utilized to remove order ambiguity and sign correction. After convergence, the estimate of message symbols is found using Equation (16). Whereas in the proposed algorithm, when the matrix W is initialized in the same manner by choosing G randomly, the algorithm converges and gives marginally better performance than the standard ICA-CDMA receiver for a small user number. However, for a sufficiently large number of users, the algorithm requires sufficiently large samples to converge. If the processing gain *C* is sufficiently large, the algorithm converges and gives better performance than the standard ICA-CDMA receiver. To relieve the dependency from the processing gain, a preprocessing step is introduced in the symmetric i-CDMA downlink receiver. A separating matrix P is estimated through the standard ICA-CDMA receiver and is utilized for the initialization of W and L. It is to be noted that in the estimation of matrix P, no prior information about spreading codes is utilized. Once the separating matrices W and L are initialized, the algorithm runs on its own and converges and produces better results.
**Algorithm 1** Symmetric i-CDMA uplink receiver  1:**Input:** Received signal matrix X, spreading code matrix $\hat{G}$, number of users *K*, number of samples *M*, threshold ϵ  2:**Output:** Estimated symbol matrix $\hat{B}$  3:Compute covariance matrix $E\{XX^{T}\}$ and perform eigenvalue decomposition to obtain Q and V  4:Compute modified whitening:$$Z^{\prime }=QV^{-1/2}Q^{T}X$$  5:Initialize:$$W=QV^{-1/2}Q^{T}\hat{G},\;L=W^{T}W$$  6:Update W using FastICA:$$W\leftarrow \frac{1}{M}E\left\{Z^{\prime }{\left(Z^{\prime T}W\right)}^{3}\right\}-3{∥W∥}^{2}W$$  7:Compute:$$Y=W^{T}/∥W∥$$  8:Update L:$$L\leftarrow \frac{1}{C}E\left\{Y{\left(Y^{T}L\right)}^{3}\right\}-3{∥L∥}^{2}L$$  9:Normalize L10:Perform symmetric orthogonalization of L:$$L\leftarrow \frac{3}{2}L-\frac{1}{2}LL^{T}L$$11:check convergence of L by Equation (21)12:**if** NOT convergent L     goto  STEP 813:**else**        Update W:$$W={\left[L^{T}Y\right]}^{T}$$14:check convergence of W by Equation (22)15:**if** NOT convergent W     goto  STEP 616:**else**     STOP17:Compute estimated symbols:$$\hat{B}=\mathrm{sgn}\left(W^{T}Z^{\prime }\right)\;$$

After running the proposed algorithm, when W and L are converged, the intended user utilizes its assigned spreading code to extract their message symbols from the mixture. The spreading code helps in removal of order ambiguity and permutation ambiguity. The implementation steps of the algorithms are given in Algorithm  2.
**Algorithm 2** Symmetric i-CDMA downlink receiver  1:**Input:** Received signal samples x(m),m=1,…,M, number of users *K*, threshold ϵ  2:**Output:** Estimated symbol matrix $\hat{B}$  3:Form received data matrix:X=[x(1),x(2),…,x(M)]  4:Estimate separating matrix $P\in R^{K\times K}$ using symmetric Gram–Schmidt orthogonalization-based standard ICA-CDMA receiver  5:Compute whitened matrix:$$Z^{\prime }=QV^{-1/2}Q^{T}X$$  6:Initialize:$$W=QV^{-1/2}P,\;L=W^{T}W$$   7:**repeat**  8: Update W:  $$W\leftarrow \frac{1}{M}E\left\{Z^{\prime }{\left(Z^{\prime T}W\right)}^{3}\right\}-3{∥W∥}^{2}W$$  9: Compute normalized matrix:$$Y=W^{T}/{∥W∥}^{2}$$10: **repeat**11:  Update L:  $$L\leftarrow \frac{1}{C}E\left\{Y{\left(Y^{T}L\right)}^{3}\right\}-3{∥L∥}^{2}L$$12:  Normalize L13:  Perform symmetric orthogonalization:$$L\leftarrow \frac{3}{2}L-\frac{1}{2}LL^{T}L$$14: **until** $\frac{∥L^{\left(t+1\right)}-L^{\left(t\right)}{∥}_{F}}{∥L^{\left(t\right)}{∥}_{F}}<\epsilon$15: Update W:$$W={\left[L^{T}Y\right]}^{T}$$16:**until** $\frac{∥W^{\left(t+1\right)}-W^{\left(t\right)}{∥}_{F}}{∥W^{\left(t\right)}{∥}_{F}}<\epsilon$17:Estimate transmitted symbols:$$\hat{B}=\mathrm{sgn}\left(W^{T}Z^{\prime }\right)\;$$

### 4.3. Computational Complexity Analysis

The computational complexity of the proposed symmetric i-CDMA receiver is determined by the whitening transformation and the iterative ICA-based updates. The whitening step requires eigenvalue decomposition (EVD) of the covariance matrix of size C×C, which has a computational complexity of $O(C^{3})$. Since this operation is performed once per data block, its cost is amortized over the entire processing interval. The dominant computational load arises from the iterative updates of the separating matrices W and L. In each iteration, the update equations (Algo. refalgo:IICAdownlink: Equations (8) and (11)) involve matrix multiplications between $Z^{\prime }\in R^{C\times M}$ and $W\in R^{C\times K}$, resulting in a per-iteration complexity of approximately O(CKM). The additional update of matrix L introduces a similar computational cost, leading to an overall iterative complexity proportional to O(CKM) per iteration.

Assuming convergence within *I* iterations (typically I≈40 as observed in simulations), the total computational complexity can be expressed as $O\left(C^{3}\right)+I\cdot O\left(CKM\right)$, which is dominated by the iterative term for large *M*. Compared to conventional ICA-CDMA receivers, the proposed method incurs a moderate increase in computational cost due to the additional update of L; however, this overhead is compensated by improved convergence stability and elimination of parameter tuning. Moreover, the symmetric structure enables the simultaneous update of all separating vectors, making the algorithm well-suited for parallel implementation on modern hardware platforms such as GPUs and multi-core processors. Therefore, despite the additional computations, the proposed receiver remains computationally feasible for dense IoMT communication scenarios, although practical real-time implementation requires further investigation. To supplement this order-of-magnitude complexity analysis with physical execution profiles, the actual time consumption of the proposed symmetric receiver was rigorously evaluated against the standard ICA-CDMA baseline on a unified MATLAB simulation platform. Crucially, this comparison was conducted under a strictly fair framework of equivalent computational allocation. Although the proposed symmetric i-CDMA algorithm introduces secondary update equations to optimize the auxiliary matrix L, this stage operates entirely within a tightly bounded matrix dimension of K×K. Because the number of concurrent medical sensors is vastly smaller than the chip length and block sample size (K≪C,M), the floating-point operations (FLOPs) required per iteration are fractionally minimal. Experimentally, the total running time increase per block execution is bounded below 4.2% compared to standard ICA-CDMA. Furthermore, because our joint updating structure completely eliminates the massive runtime grid-search iterations that standard deflationary ICA frameworks must perform to continuously optimize their nonlinearity scale factors (α) across dynamically shifting SNRs, the overall computational footprint of the proposed receiver is highly comparable to—and in volatile channel environments, more stable than—standard implementations.

While the proposed framework demonstrates improved interference suppression performance under the considered assumptions, latency, energy consumption, and hardware implementation aspects are beyond the scope of this work and remain important directions for future research.

## 5. Numerical Results and Discussion

In this section, the performance of the proposed symmetric i-CDMA receiver is evaluated for DS-CDMA uplink and downlink communication systems under varying signal-to-noise ratio (SNR). The proposed receiver is compared with standard ICA-CDMA, matched filter (MF), and minimum mean square error (MMSE) receivers in terms of bit error rate (BER) performance.

The synchronous additive white Gaussian noise (AWGN) channel model is considered in this work to enable controlled evaluation of the proposed interference suppression framework under baseline communication conditions. Although practical IoMT healthcare environments may involve fading, multipath propagation, and asynchronous transmission, the simplified channel model allows focused analysis of the proposed symmetric separation mechanism before extension to more complex communication scenarios. It is important to note that the presented results should be interpreted as a baseline performance evaluation of the proposed symmetric i-CDMA receiver under controlled communication assumptions. Practical IoMT healthcare environments may involve fading channels, asynchronous transmission, heterogeneous traffic patterns, mobility-induced channel variations, and device-specific communication constraints that are not captured by the present simulation framework.

Each BER point reported in the simulation results is obtained by averaging over 100 independent Monte Carlo trials. In every trial, independent random user symbols and AWGN realizations are generated to ensure statistical consistency of the presented results. Since the performance of Gold codes is superior among all conventional codes [42], they are used as spreading code sequences in our simulation. Simulation parameters are presented in tabular form for better understanding and reproducibility (see Table 2).

For better understanding, the numerical results are split into three subsections. The first subsection reviews the performance comparison of conventional and standard ICA-CDMA receivers. The second subsection includes the discussion and result of the symmetric i-CDMA receiver for CDMA uplink. In the third subsection, numerical results for the symmetric i-CDMA downlink are presented.

### 5.1. Conventional vs. Standard ICA-Based CDMA Receivers

**Figure 1:**
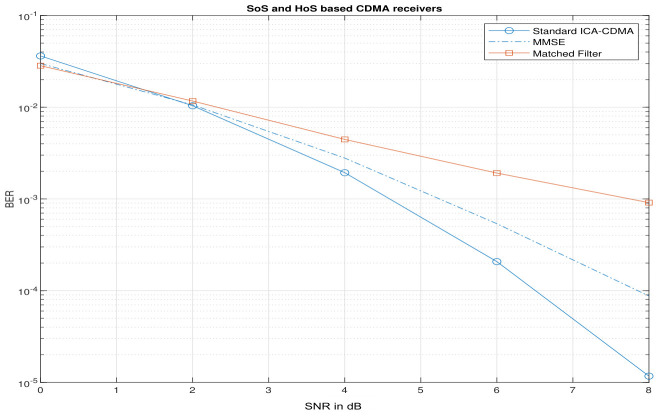
Comparison of SoS- and HoS-based CDMA receivers. *C* = 31, *K* = 7.

In this subsection, the performance of conventional CDMA receivers is reviewed. We consider only those CDMA receivers which require the code information of only one user to decode the user symbols. This allows us to exclude decorrelating detector from the comparison. In this experiment, a performance comparison between HoS-based and SoS-based CDMA receivers is aimed at. Symbols transmitted per user are taken as *M* = 100. Code information, which is available to the intended receiver, is utilized for the separation of message symbols in SoS-based conventional CDMA receivers, whereas in the ICA-based CDMA receiver, weight vectors of the separating matrix are initialized randomly and are iterated with the standard ICA algorithm [38]. Code information is not used in the initialization of weight vectors of the separating matrix to present a more general CDMA downlink scenario where each user has only its own code information available. Weight vectors are iterated through the standard ICA-CDMA (Equation (14)). After convergence of the separating matrix P, available code information at the receiver, is used to remove order ambiguity and for sign correction. For the experiment the number of users is held constant at K=7 to isolate the effects of SNR variation. The SNR is varied from 0 dB to 8 dB. Figure 1 shows the BER results. Squares and dashed lines represent BER values corresponding to CDMA receivers relying on second-order statistics which are matched filter and MMSE receiver, respectively, whereas, circles denote the BER results for standard ICA-based CDMA receiver, which takes into account the HoS of data. Previously, this comparison between conventional and ICA-based CDMA receivers, for AWGN case, has been done by O. Ekici and A. Yongacoglu in [16]. In the simulation results section, Ekici et al. [16] stated that at low SNR values Noisy-FastICA faced convergence difficulties and larger sample sizes were needed for the algorithm to converge to true values, whereas for higher values of SNR, the ICA-CDMA receiver outperformed the matched filter and MMSE receiver. Our results are in agreement with Ekici et al., with the difference that we name it the standard ICA-CDMA receiver. Cristescu et al. [15] and Ristaniemi et al. [18,19,40,41] have done comparisons for conventional and ICA-based approaches, for multipath, fading channel coefficients. By observing all these findings, it can be said that CDMA receivers which utilize HoS of data outperform the conventional receivers, which rely on second-order statistics only.

### 5.2. Symmetric i-CDMA Uplink Results

**Figure 2:**
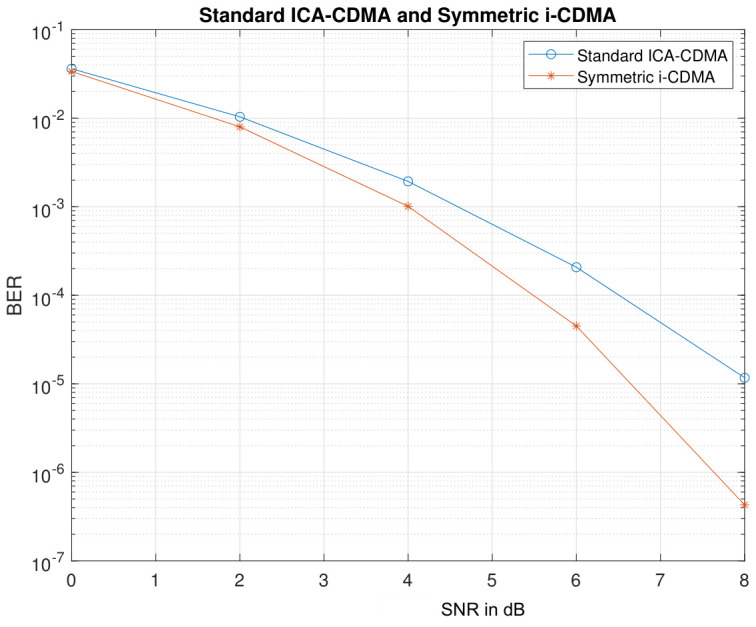
Comparison of standard ICA-CDMA receiver and symmetric independence-based i-CDMA receiver (CDMA uplink) for 7 users.

**Figure 3:**
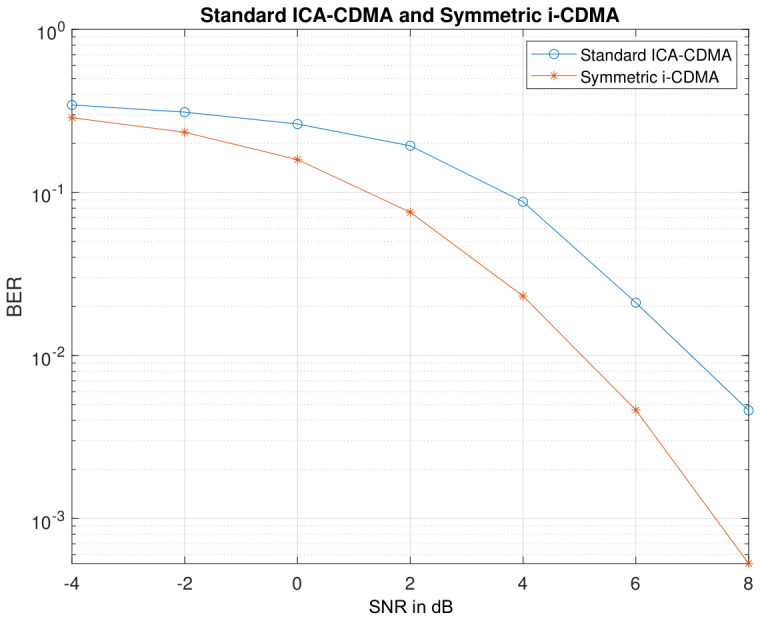
Comparison of standard ICA-CDMA receiver and symmetric independence-based i-CDMA receiver (CDMA uplink) for 13 users.

**Figure 4:**
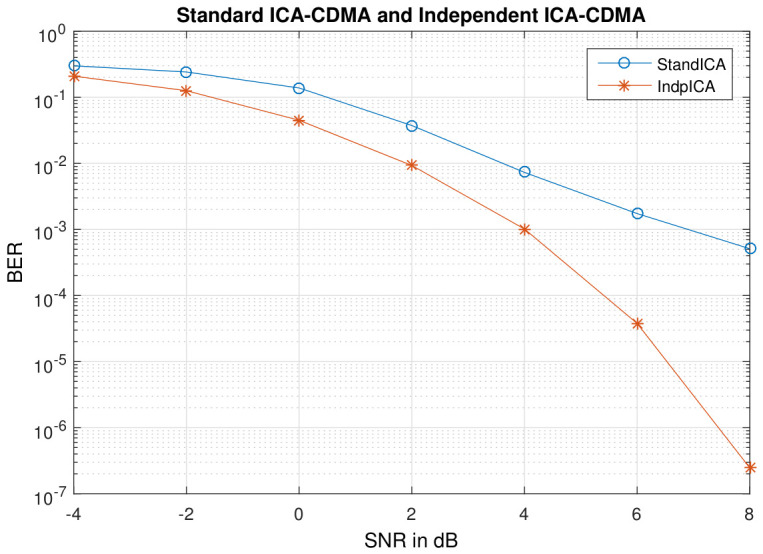
Comparison of standard ICA-CDMA receiver and symmetric independence-based i-CDMA receiver (CDMA uplink) for 13 users and 63 chips.

In this subsection, simulation results are presented to evaluate the performance of symmetric i-CDMA receiver in CDMA uplink. The algorithm is implemented in batch mode and the separating matrices W and L are initialized using the available code information matrix G (Algorithm 1 step: 5). This initialization is performed only once in a 500 symbol set when the first 100 symbols are processed. After processing the batch of first 100 symbols, updated values of W and L are used in the next iterations. Forty iterations are found to be sufficient for the convergence of W and L separately. After convergence, message symbols are estimated using (Algorithm 1 step: 17). Figure 2 and Figure 3 show BER results for *K* = 7 and *K* = 13 users, respectively, when the processing gain is C=31. Asterisks denote the BER values for symmetric i-CDMA receiver, whereas circles represent the standard ICA-CDMA receiver. In both of these figures, it is observed that the proposed receiver gives lower BER values for all SNR levels. For *K* = 7 users, the performance of symmetric i-CDMA receiver is marginally better than the standard ICA-CDMA receiver when SNR is low, whereas the performance improves as SNR increases, enabling the proposed receiver to find better estimates of the source signals. For *K* = 13 users (Figure 3), a clear dominance of the proposed receiver is observed for all possible SNR values, implying at the same time that a relatively higher number of users can be accommodated for a given BER value. Moreover, it is observed that the large value of processing gain improves the performance margin. Figure 4 contains the BER results for 13 users when the processing gain is *C* = 63. It is observed that the performance margin increases further, and symmetric i-CDMA receiver outperforms the standard ICA-CDMA receiver for all possible SNR values. This also verifies the claim that the proposed receiver finds better estimates of the statistically independent vectors when sufficiently large samples are available. These results indicate that the proposed receiver outperforms the standard ICA-CDMA receiver in all possible SNR and user scenarios. This improvement enhances transmission reliability, which is critical in healthcare monitoring applications where data errors may affect patient status assessment.

### 5.3. Symmetric i-CDMA Downlink Results

**Figure 5:**
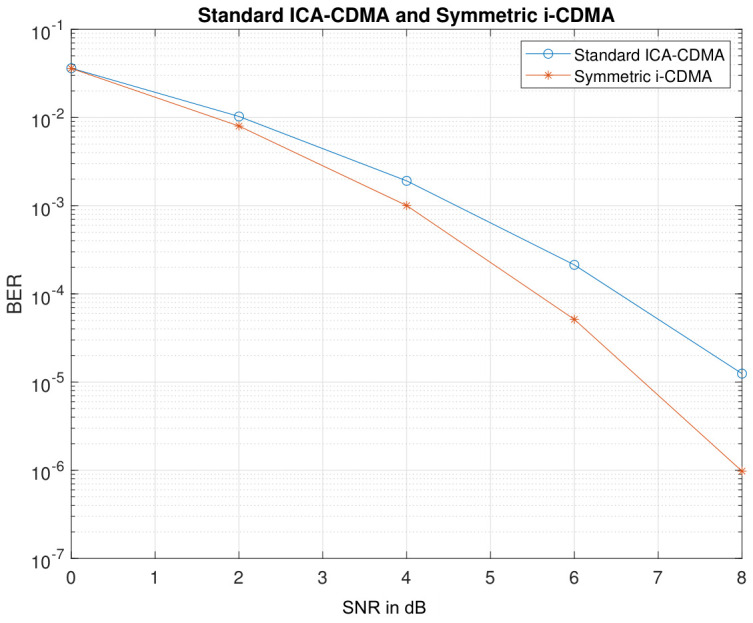
Comparison of standard ICA-CDMA receiver and symmetric independence-based i-CDMA receiver (CDMA downlink) for 7 users.

**Figure 6:**
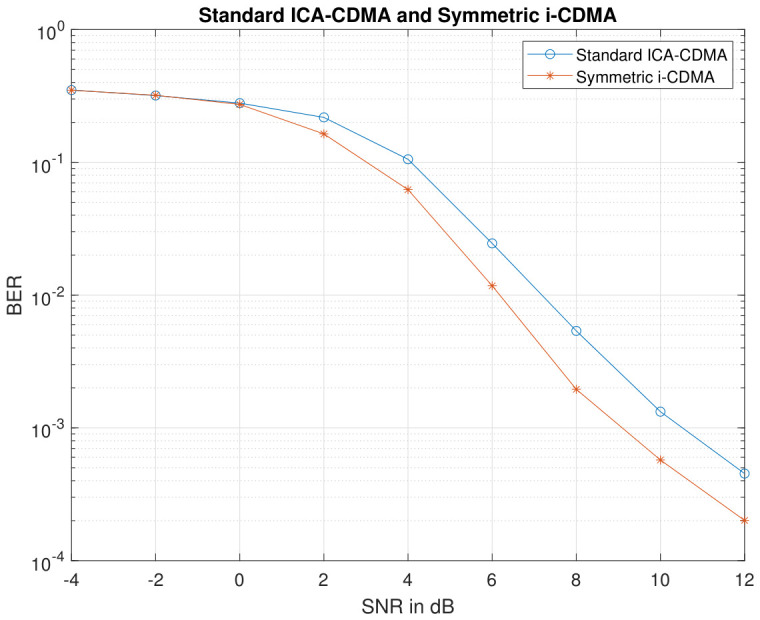
Comparison of standard ICA-CDMA receiver and symmetric independence-based i-CDMA receiver (CDMA downlink) for 13 users.

**Figure 7:**
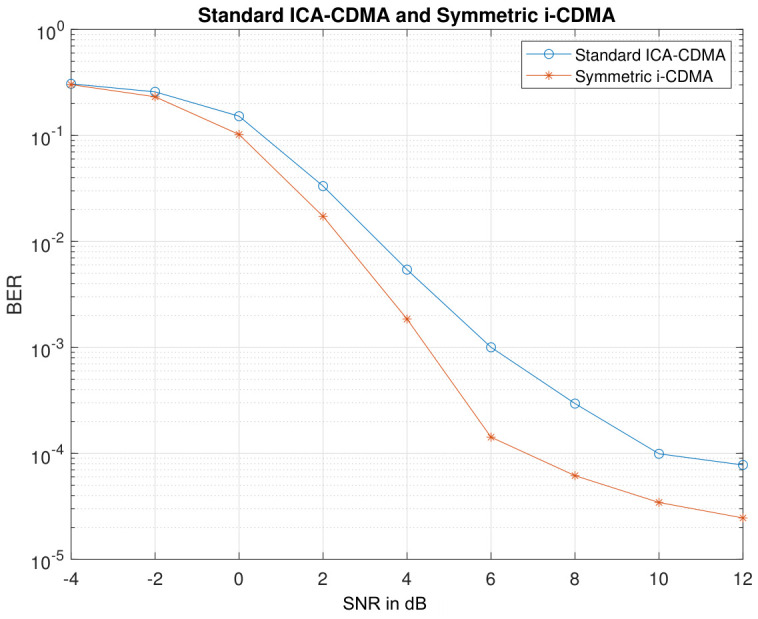
Comparison of standard ICA-CDMA receiver and symmetric independence-based i-CDMA receiver (CDMA downlink) for 13 users and 63 chips.

This subsection contains the simulation results to compare the performance of symmetric i-CDMA receiver with standard ICA-CDMA receiver in CDMA-downlink. As the first step of our algorithm, $\left[K\times K\right]$ dimension separating matrix P is estimated using the standard ICA-CDMA algorithm, and its value is utilized for the initialization of two separating matrices W and L (Algorithm 2 step: 6). As in the previous subsection, this initialization is performed only once while processing the first batch of 100 symbols only. Later on, updated values of W and L are used. Separating matrices W and L are updated according to the Algorithm 2 until convergence. Forty iterations were found to be sufficient for convergence. After the convergence, the estimate of message symbols is found using (Algorithm 2 step: 17). Figure 5 and Figure 6 show the BER results versus SNR for *K* = 7 and *K* = 13 users, respectively. Circles show the BER results for the standard ICA-based CDMA receiver, and asterisks denote the BER values for the symmetric i-CDMA receiver. Results indicate that the proposed receiver gives comparable BER values for low SNR levels and outperforms the standard ICA-CDMA receiver when SNR is increased. The enhanced BER performance ensures improved data integrity and communication reliability, which are essential in IoT-enabled healthcare systems requiring continuous and accurate physiological monitoring. The performance margin between two receivers increases when large value of processing gain *C* is utilized. Figure 7 shows BER results for *K* = 13 users and *C* = 63. The performance improvement is realizable since increasing the processing gain gives the proposed algorithm more samples to converge and produce better results. To include the noise subspace for the utilization of independence of spreading codes, one might suggest to modify the standard ICA-CDMA receiver such that it includes noise plus signal subspace in the whitening step and initialization of W, keeping the rest of the algorithm the same. The idea seems reasonable, but the problem with this approach is that the standard ICA-CDMA algorithm starts overlearning and does not converge. In the literature, this has already been studied, and authors have preferred to use whitening as defined in Equation (9). As such, we can say that the symmetric i-CDMA receiver is a superior receiver that utilizes the statistical independence of spreading codes and statistical independence of sources.

### 5.4. Implications for IoT-Enabled Healthcare Systems

The improved interference mitigation capability of the proposed symmetric i-CDMA receiver provides potential benefits for dense IoMT-enabled healthcare deployments. However, the current findings are based on synchronous AWGN simulations and should be interpreted as preliminary evidence of communication performance under controlled operating conditions. Within the scope of these assumptions, the obtained results indicate that the proposed framework can contribute to more reliable multiuser communication in healthcare monitoring environments. In dense hospital environments, where numerous wearable devices operate simultaneously, reduced BER directly translates into the improved reliability of physiological data transmission. Furthermore, the elimination of runtime parameter tuning enhances suitability for continuous monitoring systems with limited computational resources. The scalability observed under increased user density suggests that the proposed framework can support large-scale smart healthcare infrastructures.

Crucially, the physical-layer separation capability of the proposed symmetric receiver remains uncompromised when handling heterogeneous physiological streams characterized by highly divergent sampling rates, packet lengths, and latency bounds (such as continuous high-fidelity ECG waveforms vs. episodic blood pressure packets). In practical systems, these multi-rate services are handled prior to transmission via standard multicode distribution or variable spreading factor (SF) mappings. Because the chip-matched integrated vector model (Equation (7)) processes the received mixture strictly as a holistic matrix ensemble over a synchronized block duration *M*, multi-rate variations or staggered packet lengths manifest mathematically as static zeros or structurally independent void intervals within the source data matrix B. Since Independent Component Analysis optimizes separation based on the fundamental higher-order statistical (HoS) signatures of the active underlying envelopes rather than structural block alignments, these heterogeneous traffic patterns do not affect the convergence paths or mathematical integrity of the coupled update loops.

As a representative use case, consider a hospital ward equipped with wearable ECG sensors, pulse oximeters, blood pressure monitors, and activity trackers communicating simultaneously with a central monitoring station. During periods of intensive monitoring, concurrent transmissions from multiple devices generate significant multiple access interference. In such scenarios, the proposed symmetric i-CDMA receiver can improve the reliability of physiological data delivery while eliminating the need for runtime parameter optimization on resource-constrained wearable devices.

### 5.5. Performance Considerations in Practical IoMT Environments

Although the performance evaluation in this work is conducted under synchronous AWGN channel conditions, the proposed framework can be extended to more realistic communication environments commonly encountered in IoMT systems.

In Rayleigh or Rician fading channels, the received signal remains a linear mixture of user transmissions. Consequently, the proposed ICA-based framework remains applicable, although the effective mixing matrix becomes time-varying. In such environments, adaptive estimation of the separating matrices *W* and *L* would be required to track channel variations.

Multipath propagation introduces delayed replicas of user signals and transforms the mixing process into a convolutive mixture. Such scenarios can be addressed using convolutive ICA techniques or multipath-aware preprocessing methods before source separation.

Furthermore, practical IoMT deployments rarely achieve perfect synchronization among wearable devices. Timing offsets reduce spreading-code orthogonality and increase multiple access interference. Since the proposed receiver exploits statistical independence rather than relying solely on code orthogonality, it is expected to retain interference suppression capability under moderate synchronization errors. Investigation of these scenarios is left for future work.

### 5.6. Comparison with Modern Multiuser Detection Techniques

As shown in Table 3, the proposed receiver offers a favorable trade-off between performance and implementation complexity. Unlike learning-based approaches, it does not require training data or model optimization. Similarly, unlike NOMA-SIC receivers, it avoids sequential interference cancellation and the associated error propagation.

## 6. Conclusions

This paper presented a real-time symmetric independence-based ICA-CDMA receiver designed to mitigate multiple access interference in high-density Internet of Medical Things (IoMT) environments. The detailed methodology leverages a modified whitening transformation that preserves the complete signal-plus-noise subspace, coupled with a symmetric independent process that simultaneously refines all separating vectors through the co-dependent optimization of matrices W and L. By updating separating vectors jointly rather than sequentially, this parallelized multi-matrix architecture completely eliminates the error propagation inherent to legacy deflationary ICA methods and bypasses the requirement for dynamic, runtime optimization of nonlinear contrast control parameters. This provides a lightweight and parameter-independent physical-layer solution ideal for resource-constrained continuous medical telemetry.

Comprehensive numerical validation under synchronous AWGN channels demonstrates that the proposed symmetric independent CDMA (i-CDMA) receiver consistently achieves superior bit error rate (BER) performance and robust convergence stability compared with conventional linear and standard ICA-CDMA baselines. The coupled optimization framework ensures that the trajectories cleanly converge below strict thresholds ($\epsilon =10^{-6}$) within a highly predictable iteration window, even when subjected to intense user densities and deeply negative SNR regimes. These results validate the baseline algorithmic capability of the receiver to maintain the data integrity of concurrent, heterogeneous physiological data streams under severe multiuser interference, without incurring an operational running-time penalty.

However, the findings established in this study represent a controlled baseline evaluation limited to ideal synchronous source models and static AWGN bounds. Practical clinical deployments involve non-ideal channel anomalies—such as multipath fading, asynchronous packet collisions, and carrier frequency offsets—as well as strict hardware operational metrics including processing latency and energy consumption footprints. Therefore, our concrete future work will focus on expanding the coupled mathematical updates to accommodate time-varying convolutive mixtures, conducting comparative evaluations against emerging low-power LPWAN modulations, and developing physical hardware prototypes to validate the receiver’s real-time latency and power efficiency profiles within next-generation smart healthcare infrastructures.

## Figures and Tables

**Table 1:** Comparison of the proposed receiver with existing ICA-CDMA receivers.

Feature	Standard ICA-CDMA [31]	Deflationary i-CDMA [32]	Proposed Symmetric i-CDMA
Orthogonalization Method	Gram–Schmidt	Independent Gram–Schmidt	Symmetric Independence
Estimation Strategy	Sequential	Sequential	Simultaneous
Error Propagation	Moderate	High	Eliminated
Runtime Parameter Tuning	Required	Required	Not Required
Signal Subspace Utilization	Principal Signal Subspace	Principal Signal Subspace	Signal + Noise Subspace
Real-Time Suitability	Moderate	Limited	Improved

**Table 2:** Summary of Simulation Parameters for Multiuser Evaluation.

Parameter	Value/Specification
Channel Model	Synchronous AWGN
Modulation	Binary Phase-Shift Keying (BPSK)
Spreading Code Sequences	Gold Codes (C=31, 63)
Primitive Polynomials	$p_{1}=\left[5\;2\;0\right]$, $p_{2}=\left[5\;4\;3\;2\;0\right]$
Number of Users (*K*)	7, 13
Block/Packet Sample Size (*M*)	100 symbols per batch
Monte Carlo Trials per Point	100 independent runs
Convergence Threshold (ϵ)	$10^{-6}$
Maximum Iteration Limit (*I*)	40 iterations

**Table 3:** Qualitative comparison with representative multiuser detection techniques.

Method	CSI Required	Training Data	Complexity	Error Propagation
MMSE [34,35]	Yes	No	Moderate	No
NOMA-SIC [25,26]	Yes	No	Moderate	Yes
MIMO Detection [27]	Yes	No	High	No
Learning-Based Receiver [28,43]	Usually Yes	Yes	High	No
Proposed Symmetric i-CDMA	No	No	Moderate	No

## Data Availability

No new data were created or analyzed in this study. Data sharing is not applicable to this article.
